# Alanyl-Glutamine Restores Tight Junction Organization after Disruption by a Conventional Peritoneal Dialysis Fluid

**DOI:** 10.3390/biom10081178

**Published:** 2020-08-13

**Authors:** Maria Bartosova, Rebecca Herzog, David Ridinger, Eszter Levai, Hanna Jenei, Conghui Zhang, Guadalupe T. González Mateo, Iva Marinovic, Thilo Hackert, Felix Bestvater, Michael Hausmann, Manuel López Cabrera, Klaus Kratochwill, Sotirios G. Zarogiannis, Claus Peter Schmitt

**Affiliations:** 1 Division of Pediatric Nephrology, Center for Pediatric and Adolescent Medicine, University Hospital Heidelberg, 69120 Heidelberg, Germany; Maria.Bartosova@med.uni-heidelberg.de (M.B.); levai.eszter@semmelweis-univ.hu (E.L.); jeneihanna94@gmail.com (H.J.); conghui.zhang@med.uni-heidelberg.de (C.Z.); Iva.Marinovic@med.uni-heidelberg.de (I.M.); sotiris.zarogiannis@med.uni-heidelberg.de (S.G.Z.); 2 Christian Doppler Laboratory for Molecular Stress Research in Peritoneal Dialysis, Department of Pediatrics and Adolescent Medicine, Medical University of Vienna, 1090 Vienna, Austria; rebecca.herzog@meduniwien.ac.at (R.H.); klaus.kratochwill@meduniwien.ac.at (K.K.); 3 Division of Pediatric Nephrology and Gastroenterology, Department of Pediatrics and Adolescent Medicine, Comprehensive Center for Pediatrics, Medical University of Vienna, 1090 Vienna, Austria; 4 Kirchhoff Institute for Physics, Heidelberg University, 69120 Heidelberg, Germany; d.ridinger@mailbox.org (D.R.); hausmann@kip.uni-heidelberg.de (M.H.); 5 MTA-SE, Pediatrics and Nephrology Research Group, 1083 Budapest, Hungary; 6 1st Department of Pediatrics, Semmelweis University, 1083 Budapest, Hungary; 7 Immunology and Cellular Biology Department, Molecular Biology Centre Severo Ochoa, 28049 Madrid, Spain; gtirma@gmail.com (G.T.G.M.); mlcabrera@cbm.csic.es (M.L.C.); 8 General, Visceral and Transplantation Surgery, Heidelberg University, 69120 Heidelberg, Germany; Thilo.Hackert@med.uni-heidelberg.de; 9 German Cancer Research Center (DKFZ), 69120 Heidelberg, Germany; f.bestvater@dkfz.de; 10 Department of Physiology, Faculty of Medicine, University of Thessaly, 41500 Larissa, Greece

**Keywords:** peritoneal dialysis, tight junctions, paracellular transport, alanyl-glutamine

## Abstract

Understanding and targeting the molecular basis of peritoneal solute and protein transport is essential to improve peritoneal dialysis (PD) efficacy and patient outcome. Supplementation of PD fluids (PDF) with alanyl-glutamine (AlaGln) increased small solute transport and reduced peritoneal protein loss in a recent clinical trial. Transepithelial resistance and 10 kDa and 70 kDa dextran transport were measured in primary human endothelial cells (HUVEC) exposed to conventional acidic, glucose degradation products (GDP) containing PDF (CPDF) and to low GDP containing PDF (LPDF) with and without AlaGln. Zonula occludens-1 (ZO-1) and claudin-5 were quantified by Western blot and immunofluorescence and in mice exposed to saline and CPDF for 7 weeks by digital imaging analyses. Spatial clustering of ZO-1 molecules was assessed by single molecule localization microscopy. AlaGln increased transepithelial resistance, and in CPDF exposed HUVEC decreased dextran transport rates and preserved claudin-5 and ZO-1 abundance. Endothelial clustering of membrane bound ZO-1 was higher in CPDF supplemented with AlaGln. In mice, arteriolar endothelial claudin-5 was reduced in CPDF, but restored with AlaGln, while mesothelial claudin-5 abundance was unchanged. AlaGln supplementation seals the peritoneal endothelial barrier, and when supplemented to conventional PD fluid increases claudin-5 and ZO-1 abundance and clustering of ZO-1 in the endothelial cell membrane.

## 1. Introduction

Peritoneal dialysis (PD) is a cost-effective, life-saving renal replacement therapy for an increasing number of chronic kidney disease patients worldwide. It has significant advantages regarding early patient outcome and quality of life as compared to hemodialysis patients [1,2,3]. Overall, patient outcome is still poor [4,5] and PD usage is limited to a minority of patients in most countries [6,7]. Next to infectious complications, mainly bacterial peritonitis, the major disadvantage of PD is the limited toxin and fluid removal together with peritoneal protein leakage [4,6,8]. Several studies demonstrated that peritoneal protein losses predict worse outcome, i.e., cardiovascular events [9], peritonitis [10] and death [11,12], while the impact on technique survival is still uncertain [13]. Conventional PD fluids (CPDFs), which contain high concentrations of glucose and toxic glucose degradation products (GDP), have an acidic pH and unphysiological high concentrations of lactate. The highly unphysiological composition of PD fluids triggers severe peritoneal membrane transformation [14] and results in ultrafiltration decline and eventually PD failure. GDPs are rapidly absorbed and increase systemic advanced glycation end product concentrations [15,16]. Double-chamber PD fluids separate the glucose from the buffer compound, which is either lactate or bicarbonate, have a physiological pH and contain less GDPs, but still confer major peritoneal toxicity and rapidly transform the peritoneum [17]. Whether better long-term peritoneal membrane preservation [18,19] and superior preservation of residual renal function results in better PD patient outcome is still uncertain [20,21,22].

An alternative approach to reduce PD associated local and systemic toxicity and to maintain and improve peritoneal membrane transport characteristics is the addition of protective compounds to PD fluids. Alanyl-glutamine (AlaGln) restores mesothelial cell stress response and immunocompetence in vitro [23,24] and in rodents [25]. Addition of 8 mM AlaGln to CPDF during a single 4 h dwell in patients on chronic PD suggested restoration of peritoneal cellular stress responses, attenuation of sterile inflammation, and improved peritoneal host defense [26]. A subsequent randomized crossover study, using 8 mM AlaGln supplemented double chamber PD fluid over 8 weeks demonstrated higher CA-125 effluent concentrations, a marker of mesothelial cell viability, improved ex vivo (LPS and Pam_3_Cys) stimulated IL-6 release from effluent cells, and significantly improved peritoneal membrane semipermeability, i.e., higher dialytic removal of phosphate, uric acid, and potassium together with 20% lower peritoneal protein leakage [27].

Based on experimental studies [28,29] and mathematical modelling [30,31] the peritoneal endothelium is considered the key barrier limiting small and large solute and fluid removal [32,33,34]. In a global AQP-1 knock out mouse model, 50% of PD fluid-related water removal has been attributed to AQP-1 channels [35], while the molecular mechanisms of the remaining water and of the solute removal is uncertain. Ex vivo studies in sheep and human parietal peritoneum have shown that the transperitoneal permeability is reduced by vasogenic factors, such as epinephrine and endothelin-1, and that amiloride, a sodium channel blocker, has the same effect. Peritoneal permeability is defined by transcellular and paracellular pathways, with the latter being the predominant in cases of leaky membranes like the peritoneum [36,37,38]. Tight junctions (TJ) are the key paracellular barrier components of adjacent epithelial and endothelial cells, and control paracellular but also transcellular transport properties of the barrier indirectly. In endothelial monolayers, claudin 5 (CLDN5) is currently considered the most important TJ protein, which mediates the cell-cell interaction [39]. Furthermore, a critical component of the TJs is the intracellular scaffold protein zonula occludens-1 (ZO-1) that bridges the TJ claudins to actin cytoskeleton [40,41]. Proper function of TJs is seminal for the maintenance of the physiological permeability characteristics of the endothelium.

Therefore, we hypothesized, that AlaGln acts on the peritoneal membrane barrier by modifying peritoneal tight junction components and reducing mid and large-size molecule transfer and thus improving suitability for chronic PD.

## 2. Materials and Methods

### 2.1. Cell Culture

Human umbilical vein endothelial cells (HUVEC) were purchased commercially (PromoCell, Heidelberg, Germany) and kept in endothelial cell growth medium (PromoCell, Heidelberg, Germany) with supplements and antibiotics according to the manufacturer’s instructions. The HUVEC were incubated with acidic, (pH 5.5), lactate buffered PD fluid (CPDF; CAPD StaySafe ^®^, 2.3% glucose, Fresenius Medical Care, Bad Homburg, Germany), with high glucose degradation product content and with neutral pH, low glucose degradation product content, lactate buffered PD fluid (LPDF; Balance ^®^, 2.3% glucose, Fresenius Medical Care, Bad Homburg, Germany), and culture medium as control. Unspecific cytotoxicity was ruled out by lactate dehydrogenase (LDH) measurements in the supernatant photometrically in central laboratory (Analysezentrum, Heidelberg University Hospital, Heidelberg, Germany). Glucose concentration and calcium content (1.75 mmol/L) were the same in both PD fluids. An alanyl-glutamine dose-response curve was established, for further experiments, pharmacological dose of 8 mM was used, as established previously [23].

### 2.2. Transendothelial Resistance Measurements

To establish an in vitro model of HUVEC monolayer, a cell suspension (5 × 10^4^ cells/cm^2^) was seeded and cultured on a polyester mesh (Transwell, 0.4 µm pore size, 12-well type; Costar, MA, USA) under normal culture conditions. The inner and outer chambers of the Transwell were filled with 0.2 mL and 1 mL culture medium, respectively. Transendothelial electrical resistance (TER) was measured daily using an EVOM volt/ohm meter with STX-2 electrodes (World Precision Instruments, Sarasota, FL, USA). The electrodes were inserted into both ends of the mesh. An alternating current of less than ±20 µA was applied between the electrodes at a frequency of 12.5 Hz. The resistance of each monolayer was multiplied by the effective surface area (0.33 cm^2^) to obtain the electrical resistance of that monolayer [Ω cm^2^]. To calculate the normalized TER of each monolayer, background TER of a blank polyester mesh was subtracted from the TER of the respective cell monolayer. The treatment was initiated, when monolayer was formed as demonstrated by a plateau in the TER (3 days post-seeding), and the baseline TER was > 10 Ω cm^2^. In all experiments described below, HUVEC were used maximally up to the 6th passage, as recommended by the manufacturer. The data are presented as percent change of the cells cultured in culture conditions without AlaGln.

### 2.3. Paracellular Dextran Transport Assessment

Paracellular permeability of HUVEC monolayers was determined by measuring changes in the concentrations of fluorescein isothiocyanate (FITC) labeled dextrans of different sizes (10 kDa and 70 kDa, all obtained from Sigma-Aldrich, St. Louis, MO, USA) in the outer transwell compartment. Each FITC-labeled dextran was added to the inner compartment of the chamber at a final concentration of 1 mg/mL. An equimolar amount of unlabeled dextran was added to the lower compartment of the polyester mesh system to maintain an isotonic condition. At 4 h after the addition of a molecular marker, a 10 µL volume of each sample was collected from both sides of the chamber. A calibration curve was established from the FITC dextran stock solution diluted with cell culture medium. After initial 1: 40 dilution of the stock solution (1 mg/mL to 25 µg/mL), an additional five steps in 1: 2 dilutions were established (calibration curve contained following points: 25 µg/mL, 12.5 µg/mL, 6.25 µg/mL, 3.12 µg/mL, 1.56 µg/mL, and 0.78 µg/mL). A total of 10 µl of sample from basolateral compartment was diluted 1:10 with cell culture media. Differences in the calibration curve due to matrix effects from different treatment solutions (culture media or PD fluids) were excluded prior to experiments. All samples were pipetted on one black 96 well plate (Greiner, Nürtingen, Germany) and were assessed using a fluorescence spectrophotometer (F-2000; Hitachi, Tokyo, Japan) at an excitation wavelength of 490 nm and an emission wavelength of 520 nm. A calibration curve was established and used for the calculation of the amount of FITC dextran, which was transported from the upper to the lower compartment. The data are presented as percent change of the cells cultured in culture conditions without AlaGln.

### 2.4. Western Blot

Cells were lysed with whole cell lysis buffer (10 mM Tris, 150 mM NaCl, 0.5% TritonX-100, 0.1% SDS, protease inhibitor). Equal amounts of 30 µg of total protein of each cell lysate were diluted with 4× loading buffer (containing 4% β-mercaptoethanol). Proteins, dependent on their expected size, were separated in a 10% or 12% polyacrylamide gel at 200 V for 45 min. The transfer of the protein onto a polyvinylidenfluoride membrane was performed in a Transblot Cell (Bio-Rad Laboratories, Munich, Germany) at 105 V for 90 min. The membrane was blocked with blocking buffer (3% bovine serum albumin, 5% milk) for 1 h at room temperature followed by incubation with specific antibodies against ZO-1 (LS-B5625, Life Span Biosciences, 1: 500) and CLDN5 (clone 4C3C2, ThermoFischer Scientific, 1: 500) overnight at 4 °C on a shaker. The membrane was rinsed once and washed three times for 5, 10 and 15 min with 0.05% Tris-buffered saline with Tween 20 (TBS-T), then incubated for 1 h at room temperature with the secondary antibody (anti rabbit horseradish peroxidase conjugated, 1:3000). Following three further washing steps with 0.05% TBS-T, enhanced chemiluminescent signal detection was performed. Equal loading of protein was assessed by re-probing the membrane for glyceraldehyde 3-phosphate dehydrogenase (GAPDH, H86504M, Meridian Life Science 1: 20,000) for 30 min. The signals were scanned and quantified densitometrically using Image Lab Software ^®^ (Bio-Rad, Hercules, CA, USA).

### 2.5. Mouse Model

All animal procedures were approved by the institutional animal care committee of the Molecular Biology Center Severo Ochoa-Spanish National Research Council (CSIC) and conducted in accordance with institutional guidelines that comply with the Directive of the European Parliament and of the Council on the Protection of Animals Used for Scientific Purposes. A total of 42 female mice (C57BL/6) aged 14 weeks were purchased from Charles River (Barcelona, Spain) and were maintained in conventional conditions with ad libitum food and water. Each animal’s state of health was checked daily, one animal had to be excluded due to catheter malfunction. Peritoneal catheters connected to a subcutaneous mini-access port (Access Technologies, Skokie, IL, USA) for instillation of the fluids were implanted as previously described [42]. Following one week of recovery, mice were administered 2 mL fluid twice per day, 6 times a week for 7 weeks. Mice were exposed to single-chamber PDF (StaySafe^®^, 4.25% glucose, Fresenius Healthcare, Bad Homburg, Germany, CPDF) or to physiological saline (0.9% NaCl) as control. For AlaGln supplementation mice were administered to the same fluids with addition of 8 mM AlaGln (Dipeptiven, Fresenius Kabi, Bad Homburg, Germany). CPDF and AlaGln containing solutions were freshly prepared each day and used within 12 h and were warmed up to 37 °C prior to instillation. On the last day of the experiment all mice received 2 mL group-specific fluid and sacrificed after a 30 min dwell. The effluent was removed after a small incision of the peritoneal wall and completely recovered via a capillary. Parietal peritoneal biopsies were collected from the abdominal side contralateral to the catheter insertion site.

### 2.6. Immunohistochemistry

To analyze peritoneal fibrosis, parietal peritoneum was fixed in Bouin solution (containing picric acid) and paraffin embedded. Slices of 5 µm thickness were then stained with Masson’s Trichrome staining and at least 25 measurements of peritoneal thickness per tissue sample were performed. Additionally, in order to analyze peritoneal fibrosis by formation of collagen fibers, slices of parietal peritoneum fixed in formalin were stained with Sirius Red, a histological stain specific for collagen I and collagen III. Immunohistochemistry was performed on formalin fixed paraffin embedded tissue sections. Dewaxed and rehydrated tissue sections underwent heat-induced antigen retrieval performed in a pressure cooker, using the antigen retrieval citrate buffer, pH 6 (Dako, Agilent, Santa Clara, CA, USA). Endogenous peroxidase activity was blocked by universal blocking solution (Dako, Agilent, Santa Clara, CA, USA). The primary antibodies (rat anti mouse CD31 from Dianova, Germany, and rabbit CLDN5 and ZO-1, both from Thermofischer Scientific, Waltham, MA, USA) were applied overnight at 4 °C in background reducing diluent (Dako Agilent, Santa Clara, CA, USA). Incubation with biotinylated secondary reagents (Vector Laboratories, Burlingame, CA, USA) for 30 min was followed by the incubation with the Avidin Biotin Complex reagent (Vector Laboratories, Burlingame, CA, USA) for 15 min at RT. 3′3 diaminobenzidine (Dako Agilent, Santa Clara, CA, USA) was used for detection. Diluent without primary antibody was used as negative control. Cell nuclei were counterstained with hematoxylin Harris (Leica, Wetzlar, Germany).

### 2.7. Digital Image Analysis

Quantification of microvessel density was performed on CD31 stained tissues, submesothelial area reaching to the muscle was annotated as region of interest (ROI) and microvessel algorithm v1 (Image Scope, Leica, Wetzlar, Germany) was used. Fibrosis was analyzed on Sirius red staining as described previously [43] after validation of thresholds, collagen deposition is expressed in percentage submesothelial positive area. Immunohistochemical stainings were evaluated using the Aperio Positive Pixel Count Algorithm (version 9) for quantification of the amounts of positive pixels per scanned virtual slide. Mesothelial cell layer and arterioles were defined as ROI, excluding surrounding tissue and lumen, respectively. Intensity ranges for weak, medium and strong signals and negative pixels were validated specifically for CLDN5. Positivity was calculated as total number of positive pixels divided by total number of pixels in ROI area express in percentage. All tissue samples for one marker were stained in one run.

### 2.8. Immunofluorescence Staining

HUVECs were grown on transwell inserts (Corning, Thermofischer Scientific, Germany) until confluence and consequently treated with PD fluid with or without 8 mM AlaGln for 5 h. They were fixed in ethanol at −20 °C for 5 min, permeabilized (0.5 % TritonX in PBS) and blocked (5% bovine serum albumin in PBS) for one hour at room temperature. Incubation with the primary antibody was performed overnight at 4 °C. Secondary, fluorescently labelled antibody against the host species of the primary antibody was added for one hour. Nuclei were 4′,6-diamidin-2-phenylindol (DAPI) stained and filters were imaged with a confocal microscope (Leica TCS SP8) using a 63× oil objective (NA = 1.3). A total of 21 z-stacks with a z-spacing of 3 µm were taken. The microscope settings were the same for all samples. The grey scale images were processed with FIJI/ImageJ [44] to z-projections with maximal intensity and the signal was quantified at 5 randomly selected areas of the same size.

### 2.9. Single Molecule Localization Microscopy (SMLM)

The non-commercial SMLM system [45] is guarded from external influences by thermomechanical stabilization (±10^−2^ °C), temperature monitoring of the measurement environment and individual water cooling of all optical elements [46]. To prevent possible effects of thermal expansion, the measurements started at about one hour after laser start-up when thermal equilibrium was reached. The microscope is equipped with a variable beam expander 10BE03-2-8 (Standa Ltd., Vilnius, Lithuania), a Flat-Top-Profile forming optics, PiShaper (AdlOptica GmbH, Berlin, Germany), a 100×/NA 1.46 oil plan apochromatic objective lens (Carl Zeiss Microscopy, Göttingen, Germany) and four lasers: 405, 491, 561, and 642 nm with maximal laser powers of 120, 200, 200, and 140 mW, respectively. For our experiments, only the 561 nm laser was used. It was controlled through the “Omicron Control Center” program. The fluorescent light was recorded by an iXon Andor Ultra EMCCD camera (Andor Technology, Belfast, Northern Ireland) (80 nm/px, EM-gain set to 100), after being separated from the illumination light with an appropriate filter. To ensure comparable measurements, an automatized protocol for image acquisition was used: After a 10 s flash at 150 mW, most fluorophores are set into a reversible bleached state. Then, 2000 images (100 ms integration time, at 150 mW) were recorded and stored as a 16-bit grey-scale * tiff image stack. In addition to the SMLM data stack, a widefield image of the relevant specimen region was recorded.

### 2.10. SMLM Data Analysis

The SMLM data were analyzed with an in-house program package based on python scripts. The procedures applied correspond to the MATLAB based software [47,48]. To reduce noise, a threshold of 3 was used and the first 30 frames of each time-stack were discarded. Following visual inspection, masks were interactively determined in order to limit analyses to membrane areas of neighboring cells. The programs detect the position of the blinking dye molecules, use a 2D Gaussian to calculate their position and compile a matrix containing the signal amplitude, the x-, y- coordinates and the corresponding errors. Based on this matrix, relative distance distribution histograms (0–200 nm) for Ripley´s structuring analysis [47], signal-count boxplots, and pointillistic images of the ZO-1 stained junction area between two endothelial cells were created. ZO-1 molecule density was defined as molecules detected per nm^2^.

### 2.11. Statistics

In vitro data are from at least four independent sets of experiments, all conditions were studied in duplicates in each of these experiments. Western blots were obtained from three independent experiments. AlaGln effect is shown as a percentage of the corresponding treatment group without AlaGln supplementation. Student *t*-test or one-way ANOVA and Sidak´s correction for multiple testing was used to compare AlaGln to respective AlaGln free group. All in vitro data are presented as mean ± SD. Histological findings in mice were tested for normal distribution (Shapiro-Wilk test and graphically) and are presented as mean ± SD or as median and interquartile range (IQR). Non-parametric tests were used in case of non-Gaussian distribution. Pearson and Spearman correlations were calculated as appropriate for parametric and a not parametric data, respectively. In all statistics, two sided tests were used and *p* < 0.05 was considered significant.

## 3. Results

### 3.1. AlaGln Increases Transepithelial Resistance and Reduces 10 kDa and 70 kDa Transport In Vitro

**Figure 1:**
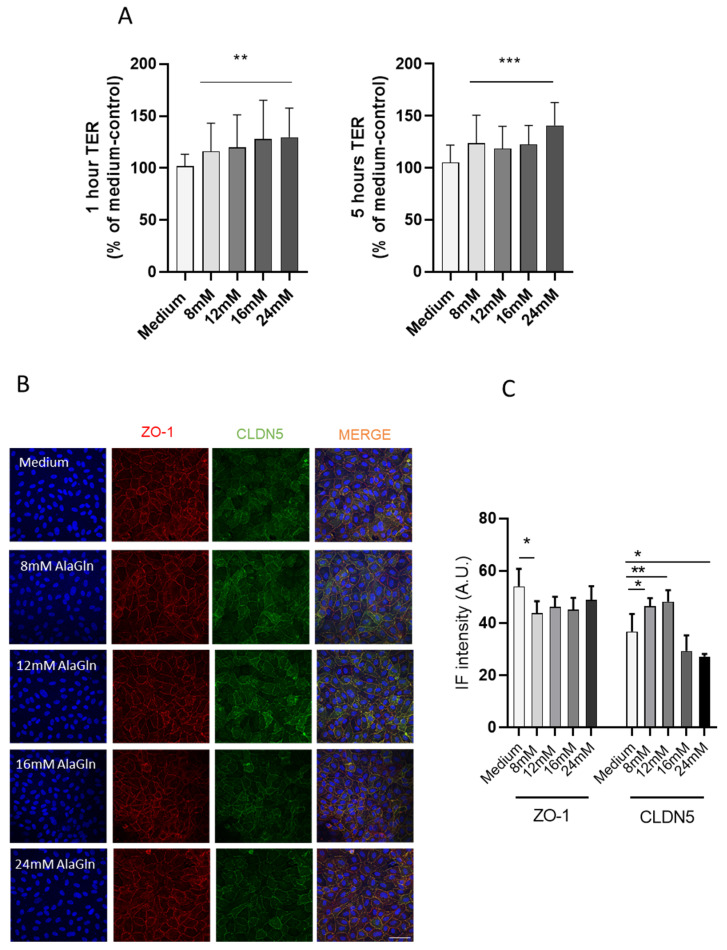
(**A**) Alanyl-glutamine (AlaGln) dose-dependently increased transendothelial resistance (TER) in human umbilical vein endothelial cells (HUVEC) incubated with cell medium (ANOVA *p* < 0.001). (**B**) gives representative zonula occludens-1 (ZO-1) and claudin-5 (CLDN5) stainings of HUVEC treated with increasing doses of AlaGln Scale bar = 50 µm, (**C**) the mean and SD values of the fluorescence signal quantification of both endothelial junction proteins. Endothelial ZO-1 abundance is decreased and CLDN5 increased with 8 mM AlaGln. A.U.= arbitrary units. * *p* < 0.05, ** *p* < 0.01, *** *p* < 0.001.

Addition of AlaGln to HUVEC culture medium resulted in a dose-dependent increase of TER. After one 1 h of incubation TER increased to (116 ± 27) %, (120 ± 31) %, (128 ± 37) %, (130 ± 28) % with 8, 12, 16, and 24 mM AlaGln compared to medium control (ANOVA *p* = 0.006; Figure 1A). After 5 h, TER increased to (124 ± 27) %, (119 ± 21) %, (122 ± 18) % and (141 ± 22) %, respectively (ANOVA *p* < 0.0001). TER reflects mainly the paracellular barrier integrity in leaky endothelia and the paracellular barrier is mainly defined by the tight junctions, of which ZO-1 is a key intracellular component [41], connected to the claudins, while CLDN5 is a key endothelial sealing junction protein [39]. ZO-1 abundance was reduced in HUVEC with AlaGln, while CLDN5 increased at currently in clinical trials used pharmacologic levels of about 8 mM and decreased with higher AlaGln concentrations (Figure 1B,C).

10 kDa and 70 kDa dextran transport across the endothelial barrier was dose dependently reduced with AlaGln. After four hours, 8 mM AlaGln added to medium decreased 70 kDa transport across the endothelial barrier (*p* = 0.03), 10 kDa transport was unchanged (*p* = 0.15; Table 1). The 24 mM AlaGln reduced 10 kDa and 70 kDa dextran transport to (32 ± 0.7) % and (61 ± 38) % of medium (*p* = 0.001/0.008). Based on these findings and since effects on cellular stress response in vitro [23,49] and on peritoneal transport in PD patients [27] were studied with 8 mM AlaGln, as higher doses of AlaGln in the clinical setting may be associated with no benefit [50], this concentration was used throughout subsequent experiments.

**Figure 2:**
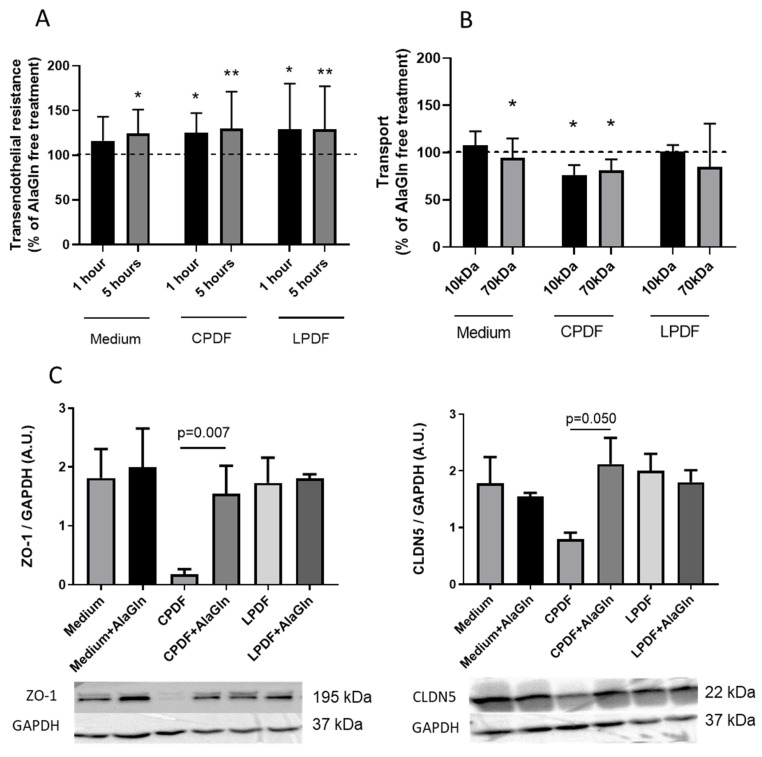
Supplementation of 8 mM alanyl-glutamine (AlaGln) to CPDF and LPDF increased electrical resistance of HUVEC after 1 and 5 h (**A**). 10 kDa and 70 kDa dextran transport was reduced with CPDF only (**B**). Reduction of ZO-1 and CLDN5 protein abundance in CPDF treated HUVEC was restored with AlaGln supplementation (**C**). Data are mean ± SD. A.U. = arbitrary units. * *p* < 0.05, ** *p* < 0.01.

Addition of 8 mM AlaGln to conventional PD fluid (CPDF) and low GDP PD fluid (LPDF) increased TER after 1 and 5 h compared to CPDF and LPDF only (Table 1, Figure 2A). Transendothelial 10 kDa and 70 kDa transport was reduced with AlaGln supplementation to CPDF (*p* = 0.02/0.04) but not when added to LPDF (*p* = 0.51/0.55; Table 1, Figure 2B).

### 3.2. AlaGln Preserves ZO-1 and CLDN5 Abundance in CPDF Treated HUVEC

Incubation of HUVEC with CPDF reduced scaffolding protein ZO-1 and endothelial sealing junction CLDN5 abundance (9.5 ± 2.2) % and (45 ± 12) % of medium control, (*p* = 0.0049/0.02), addition of 8 mM AlaGln preserved ZO-1 and CLDN5 (87 ± 23) % and (129 ± 69) % of medium, (*p* = 0.54/0.007 vs. medium/CPDF only and *p* = 0.56/0.05 vs. medium/CPDF only). Neither LPDF nor AlaGln supplemented LPDF modified ZO-1 abundance (98 ± 28) % and (105 ± 30) % of medium control; (*p* = 0.91/0.78 vs. medium control) and CLDN5 abundance (118 ± 47) % and (105 ± 25) % of medium control, (*p* = 0.83/0.61 vs. medium control; Figure 2C).

### 3.3. Junction Complex Organization is Altered after Alagln Incubation

**Figure 3:**
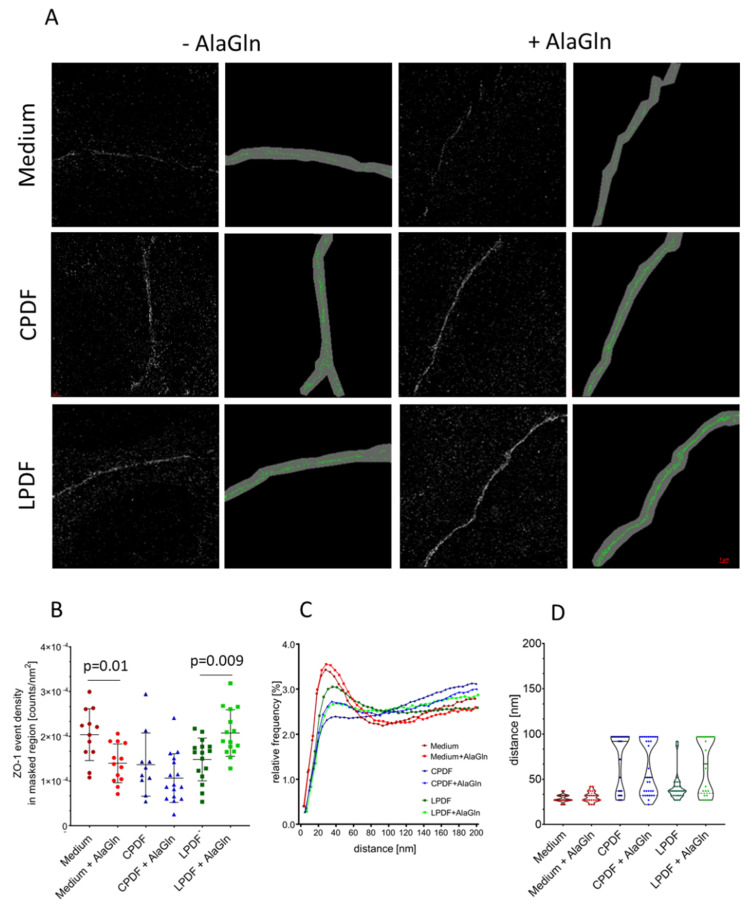
Single molecule localization microscopy was used for quantification of positioning and clustering of single ZO-1 molecules at the junction area of HUVEC, cultured on transwell filters. Representative localization images reconstructed from the loci data matrix are given in (**A**) with and without masking, mask width is 100 nm (scale bar = 1 µm). (**B**) ZO-1 molecule density for given treatments, (**C**) Ripley frequency distance curves: relative frequencies of point-to-point distances given on the x-axis (**D**) respective violin plots of the distances at which most molecules were found. Alanyl-glutamine (AlaGln) reduced the number of fluorophore signals in the membrane of endothelial cell incubated with medium and increased it with LPDF incubation (**B**). ZO-1 molecule clustering was changed in a characteristic way when AlaGln was added (**C**,**D**).

TER and permeability characteristics are defined by the quantity and the spatial organization of the intercellular junctions. We therefore performed single molecule localization microscopy (SMLM) for ZO-1 in neighboring HUVEC membrane areas (Figure 3A). In line with the immunofluorescence stainings in HUVEC (Figure 1), the number of membrane bound ZO-1 molecules per nm^2^ (density of labelling signals), as determined within the masked regions was reduced when 8 mM AlaGln, was supplemented to the medium ((1.39 ± 0.43) × 10^−4^ counts/nm^2^ with AlaGln vs. (2.04 ± 0.58) × 10^−4^ counts/nm^2^ without AlaGln, (*p* = 0.01)). It increased when AlaGln was supplemented to LPDF ((2.07 ± 0.52) × 10^−4^ counts/nm^2^ with AlaGln vs. (1.48 ± 0.48) × 10^−4^ counts/nm^2^ without AlaGln, (*p* = 0.009)). The ZO-1 number of molecules remained unchanged when 8 mM AlaGln was added to CPDF ((1.10 ± 0.55) × 10^−4^ counts/nm^2^ with AlaGln vs. (1.40 ± 0.71) × 10^−4^ counts/nm^2^ without AlaGln, (*p* = 0.23; Figure 3B)).

We then analyzed the structural organization of the ZO-1 molecules in the membrane regions according to Ripley´s distance frequency curve shapes. Peaks occurring in these curves indicate the formation of molecular clusters, a typical signature in general correlated to a given degree of functionality of membrane embedded molecules [51]. The slope of the Ripley curves at higher distances can be used as a measure for the degree of randomness of the surrounding points [52]. For AlaGln supplemented medium, the average reduction of ZO-1 molecule density was accompanied by a slight increase of clustering; the surrounding appears to be more homogenously distributed with AlaGln supplementation. AlaGln supplementation to CPDF strongly increased the clustering of ZO-1. However, the randomness of the surrounding, i.e., the slope of the curves at high distances, did not change. This means that in AlaGln supplemented medium and CPDF, the increase in cluster formation could be related to different density changes.

The addition of AlaGln to LPDF led to an increase of ZO-1 molecule density and a reduction of the ZO-1 clustering (Figure 3C,D). This behavior was observed under both data evaluation conditions, i.e., with and without masking the cellular border. Thus, clustering reflects the different resistance and transport behavior of CPDF and LPDF after addition of AlaGln that has been shown in Table 1.

### 3.4. In Vivo Effect of AlaGln Supplementation to CPDF

To validate the effect of AlaGln supplementation to CPDF in vivo, mice underwent twice daily peritoneal fluid exposure for 7 weeks. Morphological indices were similar in saline and CPDF treated mice, peritoneal cell density was lower with CPDF (Table 2). Submesothelial thickness correlated with cell number per ml effluent (ρ = 0.44, *p* = 0.005) and with submesothelial cell number per mm peritoneal section length in all animals (ρ = 0.55, *p* < 0.0001), but was unchanged with AlaGln supplementation to saline and CPDF (*p* = 0.08/0.18; Table 2). Submesothelial collagen deposition was reduced with AlaGln addition to CPDF (84%; *p* = 0.02), but not when added to saline (*p* = 0.12). Microvessel number and density were not systematically changed with AlaGln supplementation to saline (*p* = 0.93/0.66) and CPDF (*p* = 0.25/0.52). Peritoneal infiltrating cell density was high in all groups, reduced with AlaGln supplementation to saline (*p* = 0.04), and unchanged when added to CPDF (*p* = 0.24). Cell numbers per mm peritoneal section length and effluent cell counts were correlated (ρ = 0.40, *p* = 0.01), but not changed by AlaGln (*p* = 0.13/0.88 and *p* = 0.06/0.15). Fluid volume drained 30 min after injection of 2 mL of normoosmolar saline was lower than following hyperosmotic CPDF injection but unchanged with AlaGln (*p* = 0.08/0.13) in saline and the CPDF group respectively.

**Figure 4:**
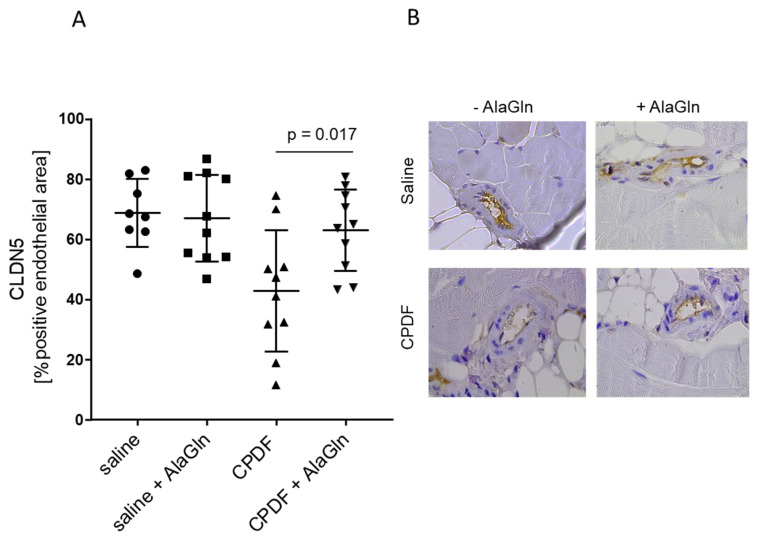
Supplementation of conventional PD fluid (CPDF) with 8 mM alanyl-glutamine (AlaGln) in mice treated for 7 weeks increased arteriolar endothelial CLDN5 abundance (left panel; means ± SD are given; *p* = 0.017 versus CPDF only) (**A**). Representative stainings of the mouse parietal peritoneum, illustrating upregulation of arteriolar endothelial CLDN5 with supplementation of AlaGln to CPDF (**B**). Scale bar = 50 µm.

Unspecific binding of peritoneal ZO-1 antibody precluded precise ex vivo quantification. CLDN5 abundance in peritoneal arterioles was lower with CPDF compared to saline (*p* < 0.001). AlaGln supplementation did not change arteriolar CLDN5 in saline group (*p* = 0.25) but increased in the CPDF group (*p* = 0.0075; Table 2, Figure 4A). Similar findings were obtained when analysis was restricted to the arteriolar endothelium. Mesothelial CLDN5 quantification was compromised by inflammatory cell coverage of the peritoneum in seven mice (4 saline, 1 saline + AlaGln and 3 in CPDF + AlaGln group). In the remaining mice, mesothelial CLDN5 positivity was lower with CPDF treatment compared to saline (*p* = 0.01). Supplementation of AlaGln did not change mesothelial CLDN5 abundance in any of the groups (*p* = 0.61/0.38).

## 4. Discussion

Limited peritoneal fluid and toxin removal together with chronic peritoneal protein losses represent an essential limitation of peritoneal dialysis, essentially contributing to poor patient outcome [4,5]. Surprisingly, little is known on the molecular mechanisms of transperitoneal water, toxin. and protein transport. Based on a global knock out mouse model, AQP-1 has been shown to mediate 50% of selective peritoneal water transport [35], whereas molecular mechanisms of solute and protein transport are unclear. The peritoneal membrane consists of a mesothelial cell monolayer, a submesothelial space composed of extracellular matrix, numerous small blood vessels and few lymphatic vessels and nerves, mostly organized in three distinct layers [53]. Peritoneal microvessel density correlates with small solute transport rates [17,54]; the vascular endothelium is considered the primary, rate limiting barrier [32,33,34]. Alterations in the endothelial barrier characteristics should significantly modify PD clearance functions. A recent clinical trial demonstrated improved semipermeability of the peritoneal membrane with AlaGln supplemented PDF, i.e., higher small solute transport rates together with reduced protein losses [27].

We now provide first experimental evidence, that AlaGln added to CPDF increases endothelial membrane abundance of sealing junction protein CLDN5, and of scaffolding TJ protein ZO-1. CLDN5 is highly expressed in endothelial cells and has sealing properties regulating the paracellular permeability of the vessels. ZO-1 is a scaffolding protein located in the inner side of the plasma membrane and through binding to claudins it mediates the communication of TJs with the cell actin cytoskeleton [40,55]. Altogether these proteins regulate endothelial barrier function. Tight junctions do not form linear structures along the neighboring endothelial cell membranes as suggested by immunofluorescence microscopy studies (Figure 1B) but cluster in modifiable functional units [56]. In our experimental setting addition of AlaGln to CPDF increased the clustering of ZO-1, the TER and reduced 10 kDa and 70 kDa dextran transport. Thus, AlaGln increases peritoneal endothelial junction expression and spatial organization and functionally reinforces the barrier function. Of note, SMLM were obtained from HUVEC grown on transwell filters to establish endothelial cell apical and basolateral polarization. In this novel setting, up to now, adequate single molecule distinction could be achieved for ZO-1, but not for CLDN5.

Supplementation of 8 mM AlaGln to culture medium reduced scaffolding ZO-1 and increased sealing protein CLDN-5 abundance, the net effect was an increased TER. In line with this, 70 kDa dextran transport was reduced as compared to control and SMLM demonstrated reduced ZO-1 molecule number in the membrane area, but preserved clustering of ZO-1. The strong suppression of endothelial cell ZO-1 abundance by CPDF is a novel finding. Reduction of ZO-1 abundance by CPDF has previously been reported in effluent mesothelial cells [57]. In intestinal epithelial cells glutamine regulates ZO-1 abundance and TER [58], and supplementation of AlaGln mitigates toxin induced loss of membrane ZO-1 and of TER [59]. Proteome analysis of mesothelial cells exposed to CPDF suggests actin cytoskeleton disruption [60], but preservation of the cytoskeleton if supplemented with AlaGln [61]. We now provide first evidence that CPDF induced loss of endothelial cell integrity and barrier function can prevented by concomitant AlaGln treatment.

To further substantiate these findings, we administered AlaGln in a mouse model of twice daily PDF exposure using CPDF, for which in vitro 10 kDa and 70 kDa dextran transport was reduced. AlaGln reduced CPDF induced collagen deposition in the submesothelial space, a finding which is in line with the results obtained in uremic rats treated once daily with 8 mM AlaGln supplemented CPDF. Submesothelial thickness, alpha smooth muscle actin positive cell density, fibronectin, and hyaluronic acid deposition were reduced [25]. In addition to these findings, we now demonstrate an increase in peritoneal endothelial CLDN5 abundance with AlaGln. The relative CLDN5 positivity in the mesothelial cell layer was similar as in the endothelium, but not increased by AlaGln supplementation, but the latter analysis was limited by major cell invasion covering the peritoneal surface of the mice. Whether peritoneal TJ abundance has an impact on peritoneal membrane transport characteristics is not yet proven. In leaky tissues TER reflects mainly the paracellular barrier, built by TJs and indirect evidence provided by electrophysiology experiments in sheep and human peritoneum suggest that its permeability is reflected by changes in the TER [36,37,38]. Based on mathematical modelling [34] and comparison of the electrophysiological monolayer characteristics of endothelial and mesothelial cells [29], the mesothelial cell layer presumably has limited barrier function as compared to the endothelial layer.

Up to now, 27 different claudins have been identified [62,63]. We focused on CLDN5, based on the abundance in the literature regarding its important role in the tightening of the endothelial barrier of vessels as of the blood brain barrier [64]. Other sealing claudins may be regulated by AlaGln. On the other hand, our data on the increased clustering of transmembrane scaffolding protein ZO-1, as well as the resistance and transport studies clearly demonstrate the functional significance of AlaGln supplementation to conventional PDF on endothelial barrier function, i.e., reduced transport of 10 kDa and 70 kDa molecules in vitro. Precise clearance measurements could not be performed in our mouse model of PD, since both saline and PD fluid exposure resulted in major cellular infiltration of the peritoneum and high effluent cell concentrations, even though all effluent cultures were sterile. Similar findings were observed previously [25] and limit the validity of this mouse model of PD. In addition, we did not study uremic mice, since stable uremia achievable in mice only corresponds to chronic kidney disease stage 3 to 4, introducing additional variance in the model without providing firm evidence. The twice daily infusion of PD fluid without subsequent drainage reflects a PD fluid exposure model rather than a PD treatment model. A putative effect of end stage renal disease per se on junction regulation by AlaGln supplemented PD fluid cannot be excluded. Another limitation of our study regards the use of human umbilical vein endothelial cells. They are a well-established model in PD research [29], but might not reflect the expression and regulation of junctions in human peritoneal capillary endothelial cells. On the other hand, we were able to reproduce the in vitro findings in parietal peritoneal endothelial cells in mice, and these findings are in line with the functional findings reported in the clinical trial [27].

Whether the sealing of the endothelial barrier by AlaGln impacts on the resistance of the peritoneum against bacterial infections is unclear. Bacteria such as *Staphylococci* use the paracellular route to cross the epithelial barriers by disruption of junctional structures [65]. Mesothelial cells isolated form diabetic rats have altered tight junction expression and reduced TER [66]; diabetic PD patients have significantly higher risks of peritonitis [67]. Supplementation of AlaGln to the PDF in the phase 2 clinical trial significantly increased circulating AlaGln concentrations and may directly impact on the endothelial barrier [27]. These findings provide a rationale for in vitro and in vivo studies to investigate whether sealing of the endothelial barrier by AlaGln has an impact on the incidence and severity of peritonitis in patients on PD.

## 5. Conclusions

AlaGln supplemented to CPDF increased peritoneal endothelial barrier function via modification of components of the TJ complex protein abundance and clustering. Our studies provide first evidence that targeting of the molecular structures defining peritoneal membrane barrier and transport function may allow to optimize PD efficacy and thus to improve PD sustainability and ultimately patient long-term outcome.

## Figures and Tables

**Table 1:** Resistance and 10 kDa and 70 kDa transport across human primary endothelial cells (HUVEC) with 8 mM AlaGln added to medium and peritoneal dialysis (PD) fluids and expressed relative to respective AlaGln-free controls.

		Medium [%]	CPDF [%]	LPDF [%]
**Resistance**	1 h	116 ± 27	125 ± 22 *	129 ± 51 *
	5 h	123 ± 27 *	130 ± 41 **	125 ± 30 **
**Transport**	10 kDa	107 ± 15	76 ± 11 *	101 ± 7
	70 kDa	86 ± 20 *	81 ± 12 *	84 ± 46

Data presented as % of treatment without 8 mM AlaGln; * *p* < 0.05, ** *p* < 0.01 vs. treatment without AlaGln. CPDF = conventional PD fluid, LPDF = low GPD PD fluid.

**Table 2:** Parietal peritoneal tissue morphology and CLDN5 abundance of mice treated with conventional PD fluid (CPDF) and saline. Two-way ANOVA was performed for effect of AlaGln intervention, followed by t-test/Mann-Whitney test for AlaGln effect within the groups.

	Saline (*n* = 10)	Saline + AlaGln (*n* = 9)	CPDF (*n* = 11)	CPDF + AlaGln (*n* = 11)	ANOVA
Peritoneal thickness [µm] (IQR)	30.1 (12, 55)	12.2 (11, 27)	32 (29, 54)	44.2 (29, 66)	0.72
Collagen submesothelial area [%]	7.1 (2.2, 19.1)	19.6 (13.1, 29.9)	18.5 (4.3, 39.2)	3.0 (1.9, 10.9) *	0.73
Microvessel density [/mm^2^]	47.8 (23, 83)	16.6 (4, 119)	33.2 (13, 89)	60.4 (35, 73)	0.39
Microvessel number [/mm section length]	1.2 (0.6, 2.7)	0.5 (0.1, 1.3)	0.7 (0.5, 4.8)	2.2 (1.1, 4.0)	0.90
Cell density [/mm^2^]	12544 (8032, 18970)	9037 * (4833, 11115)	6008 ^#^ (2861, 8885)	8801 (6863, 11642)	0.64
Cell number [/mm section length]	614 (127, 755)	132 (63, 451)	245 (92, 345)	423 (304, 542)	0.42
Drained effluent [ml]	1.3 (1.0, 1.4)	1.4 (1.3, 1.5)	2.2 (1.9, 2.5) ^##^	1.8 (1.5, 2.2)	0.45
Effluent cells [× 10^6^ cells/mL effluent]	6.9 (4.9, 11.9)	4.9 (3.0, 8.4)	9.9 (3.8, 20.1)	9.5 (4.5, 16.6)	0.11
Arteriolar CLDN5 [% pos. area]	38 ± 9	45 ± 17	24 ± 8 ^##^	32 ± 9 **	0.04
Endothelial CLDN5 [% pos. area]	69 ± 11	67 ± 14	43 ± 20 ^##^	63 ± 14 **	0.07
Mesothelial CLDN5 [% pos. area]	58 ± 28	51 ± 28	34 ± 12 ^#^	29 ± 14	0.56

*/** = *p* < 0.05/0.01 compared to AlaGln free control. #/## *p* < 0.05/0.01 versus saline control, AlaGln = alanyl-glutamine, pos. = positive.
